# Analysis of the SARS-CoV-2 inactivation mechanism using violet-blue light (405 nm)

**DOI:** 10.1128/aem.00403-25

**Published:** 2025-05-14

**Authors:** Davide Amodeo, Serena Marchi, Lia Fiaschi, Luisa Raucci, Camilla Biba, Valentina Salvestroni, Claudia Maria Trombetta, Ilaria Manini, Maurizio Zazzi, Emanuele Montomoli, Ilaria Vicenti, Gabriele Cevenini, Gabriele Messina

**Affiliations:** 1 Department of Medical Biotechnologies, University of Siena 9313, Siena, Italy; 2 Department of Molecular and Developmental Medicine, University of Siena 9313, Siena, Italy; 3 Department of Biotechnology, chemistry and pharmacy, University of Siena 9313, Siena, Italy; 4 VisMederi srl 721579 , Siena, Italy; Centers for Disease Control and Prevention, Atlanta, Georgia, USA

**Keywords:** violet-blue light, reactive oxygen species, lipid peroxidation, protein carbonylation, SARS-CoV-2 envelope

## Abstract

The study evaluated the effects of violet-blue light (VBL) on cell viability and replication, carbonylation of three structural proteins (S, E, and N) and one non-structural protein (NSP13), and direct damage to the RNA of SARS-CoV-2. The virus was exposed to increasing doses of VBL along with influenza A and B viruses to compare their susceptibility. At the highest dose (21.6 J/cm^2^), SARS-CoV-2 was significantly more susceptible to VBL than the influenza viruses, with a reduction in viral titer of 2.33 log_10_. Viral RNA did not show significant changes after exposure to VBL, as demonstrated by next-generation sequencing and real-time PCR quantification, suggesting that the inactivation process does not involve direct nucleic acid damage. To exclude the role of the culture suspension in the inactivation process, virus viability experiments were performed using different dilutions of Dulbecco’s modified Eagle’s medium (DMEM) in phosphate-buffered saline (PBS). The results indicated that the suspension medium played a secondary role in virus inactivation, as viability did not increase with increasing DMEM dilution. Subsequent tests with three different antioxidants (NAC, AsA, and SOD) at different concentrations prevented viral inactivation, from 99.99% to 85.43% (with SOD 0.003 mM). Carbonylation of S and E proteins was more pronounced when viruses were suspended in DMEM rather than PBS, although the tests demonstrated that the intrinsic properties of the viral membrane were a crucial element to consider in relation to its susceptibility to VBL.

**IMPORTANCE:**

Light-based disinfection methods are often used in combination with other cleaning methods due to their non-invasive nature, versatility, and environmental benefits. VBL is an effective approach as it induces the production of reactive oxygen species that reduce microbial viability. In this study, lipid peroxidation was identified as an important factor affecting the structural integrity and function of the viral envelope, reducing its ability to interact with host cells and consequently its ability to be infectious. The lipid envelope of SARS-CoV-2, composed mainly of glycerophospholipids and lacking cholesterol and sphingolipids, appears to be the critical factor in its susceptibility, distinguishing it from influenza viruses, which have a lipid profile richer in components that protect against oxidative stress.

## INTRODUCTION

The COVID-19 pandemic caused a global health crisis between 2020 and 2023, resulting in over 7 million deaths by October 2024 (1). Its impact on public health continues to influence not only health outcomes but also socio-economic and political contexts (2).

Health authorities identified the virus responsible for the pandemic in January 2020, initially referring to it as “Coronavirus 2019-nCoV” and subsequently officially as SARS-CoV-2 (3). It is a single-stranded, positive-sense RNA (ssRNA+) beta-coronavirus with a genome size of approximately 30 kb, which is 80% shared with SARS-CoV (4). It encodes 11 accessory proteins (ORF3a, ORF3b, ORF3c, ORF3d, ORF6, ORF7a, ORF7b, ORF8, ORF9b, ORF9c, and ORF10), 16 non-structural proteins (NSP1–16), and 4 structural proteins (Spike [S], Envelope [E], Membrane [M], and Nucleocapsid [N]) (5). Structural proteins are essential components of the SARS-CoV-2 virion, while non-structural and accessory proteins contribute to the replication of SARS-CoV-2 RNA within the cell through different mechanisms (6). In addition, coronaviruses use a variety of host factors to infect and replicate within cells, and the expression patterns of these factors are thus co-determinants of viral tropism (4). Once the large RNA genome enters the cell and begins to translate, a cytoplasmic replication cycle begins, utilizing diverse strategies to enhance viral gene expression at both transcriptional and translational levels (7).

In response to this high replicative and adaptive capacity of the virus, international health organizations had to quickly implement appropriate preventive measures to contain and control the pandemic spread of SARS-CoV-2 (8). These included preventive and mandatory isolation of suspected or infected individuals, use of hand sanitizer, covering the nose and mouth when coughing and sneezing (to reduce aerosol transmission), use of face masks, reduction of hand-to-face contact, and use of disposable gloves to avoid direct contact with surfaces (9). In addition, several disinfection techniques have been employed to inactivate SARS-CoV-2 from contaminated surfaces or air. It is widely recognized that the virus is highly susceptible to most alcohol-based hand sanitizing solutions and detergents (10), as well as physical agents like heat (11) and UV radiation (12).

Disinfection with UV light sources has been widely employed for several decades, primarily with UVC lamps (254 nm), to inactivate pathogenic microorganisms in air, on surfaces, and in water (13–15). For example, UV light sources can provide continuous decontamination of air and surface from many respiratory pathogens, including influenza viruses (16). However, the recent pandemic has boosted this scientific sector and accelerated the technological shift from mercury vapour lamps to light-emitting diodes (LEDs), promoting a transition that had been stagnant for several years and is now increasingly recognized, particularly from an environmental perspective (17–19). The combination of (i) more versatile and longer-lasting light sources (compared to traditional lamps), (ii) an unexpected global health emergency, and (iii) a new airborne pathogen brought renewed attention to the field and encouraged the scientific community to focus on wavelengths that were previously difficult to test and expensive to implement in standard disinfection procedures (20–23). Wavelengths such as far-UVC (222 nm) and violet-blue light (VBL; at 405–450 nm) gained popularity in SARS-CoV-2 studies, during and after the pandemic, due to their significant germicidal efficacy (24, 25). Furthermore, far-UVC light exhibits a restricted penetration depth in biological tissues, primarily addressing surface pathogens while leaving deeper skin layers unaffected (26). Conversely, radiation within the 405–450 nm spectrum, especially around 405 nm, penetrates tissues more easily and exhibits antimicrobial properties by activating endogenous and exogenous photoinitiators that lead to the production of reactive oxygen species (ROS) within cells (27, 28). When viruses, bacteria, and fungi are exposed to VBL, photosensitizers such as porphyrins and flavin absorb photons, leading to the formation of ROS such as singlet oxygen (^1^O_2_) and hydrogen peroxide (H_2_O_2_) (29). ROS can cause damage to vital cellular components, including nucleic acids, proteins, and membrane lipids, ultimately leading to cell death (28).

Although numerous studies have demonstrated the efficacy of VBL on SARS-CoV-2, the hypotheses put forward to describe the possible mechanism of inactivation following the virus’s exposure to radiation have so far remained inconclusive (30–32). Some of these studies focus on the possible photo-degradation of S protein as the primary cause of the virus inactivation. Other studies attribute the main source of ROS generation and oxidative stress promotion to the suspension medium in which the virus resides during the experiments (31, 33). This interpretation, however, would not fully explain the different susceptibilities of other RNA viruses to VBL (34) and, more generally, why radiation appears to reduce and/or inactivate viruses (including SARS-CoV-2), bacteria, and moulds, even in *in vivo* studies in which the experimental factors investigated, such as culture medium and suspension, are absent (35, 36).

The aim of the study was to assess the virus inactivation mechanism using VBL (at 405 nm) by evaluating: (i) the change in viral titer of SARS-CoV-2 compared to influenza A and B viruses (IAVs and IBVs) after light exposure; (ii) the replicative cycle of the viral particle and the possible presence of oxidative stress caused by radiation; (iii) qualitative and quantitative changes in the structural components of the virus (ssRNA and proteins); and (iv) the impact of the suspension medium (Dulbecco’s modified Eagle’s medium [DMEM]) on virus viability.

## RESULTS

### TCID_50_ reduction of SARS-CoV-2 and influenza A and B by VBL

The results obtained by exposing the different viruses to VBL at different doses of light energy had a significantly different impact on virus replication depending on the virus tested. As shown in Table 1, a significantly higher log_10_ reduction in TCID_50_/mL was achieved for SARS-CoV-2 compared with influenza viruses. Specifically, the TCID_50_/mL log reduction for SARS-CoV-2 was 0.41 log_10_ (SD = 0.14) after 22′30″ (dose of 5.4 J/cm^2^), 1.33 log_10_ (SD = 0.2) after 45′ (dose of 10.8 J/cm^2^), and 2.33 log_10_ (SD = 0.14) after 90′ (dose of 21.6 J/cm^2^) exposure. For IAV, the TCID_50_/mL mean reduction was almost 0 after 22′30″, 0.13 log_10_ (SD = 0.22) after 45′, and 0.16 log_10_ (SD = 0.26) after 90′. The results for IBV were nearly identical. In fact, the TCID_50_/mL log reduction was again zero after 22′30″, 0.12 log_10_ (SD = 0.25) after 45′', and 0.17 log_10_ (SD = 0.14) after 90′.

**TABLE 1:** TCID_50_/mL mean reduction of SARS-CoV-2 and influenza A and B with VBL^*a*^

Exposure time	Energy dose (J/cm^2^)	Virus	TCID_50_/mL CV (Log_10_)	TCID_50_/mL VBL exposure (Log_10_)	TCID_50_/mL reduction		
					(Log10)	Lower CI	Upper CI
22′30″	5.4	SARS-CoV-2	5.50	5.08	0.42	0.25	0.58
		IAV	8.75	8.75	0.00	0.00	0.14
		IBV	7.88	7.88	0.00	0.00	0.14
45′	10.8	SARS-CoV-2	5.49	4.16	1.33	1.01	1.66
		IAV	8.88	8.75	0.13	0.00	0.37
		IBV	7.75	7.63	0.12	0.00	0.40
90′	21.6	SARS-CoV-2	5.49	3.16	2.33	2.17	2.50
		IAV	8.75	8.59	0.16	0.00	0.46
		IBV	7.13	6.96	0.17	0.00	0.33

^*a*^ Experiments were performed in triplicate for each virus exposed to VBL. The data shown in the table for both virus control (CV) and exposed virus are the means of the results obtained. The TCID_50_/mL reduction, together with the 95% confidence interval (CI), was obtained by calculating the mean ratio of CV to virus exposed in each experiment.

Based on SARS-CoV-2 viral load reduction results, all further tests on the inactivation mechanism of SARS-CoV-2 were performed by exposing the virus to VBL for 90 min.

### Impact of VBL on SARS-CoV-2 dsRNA and N/S protein expression and localization

**Fig 1:**
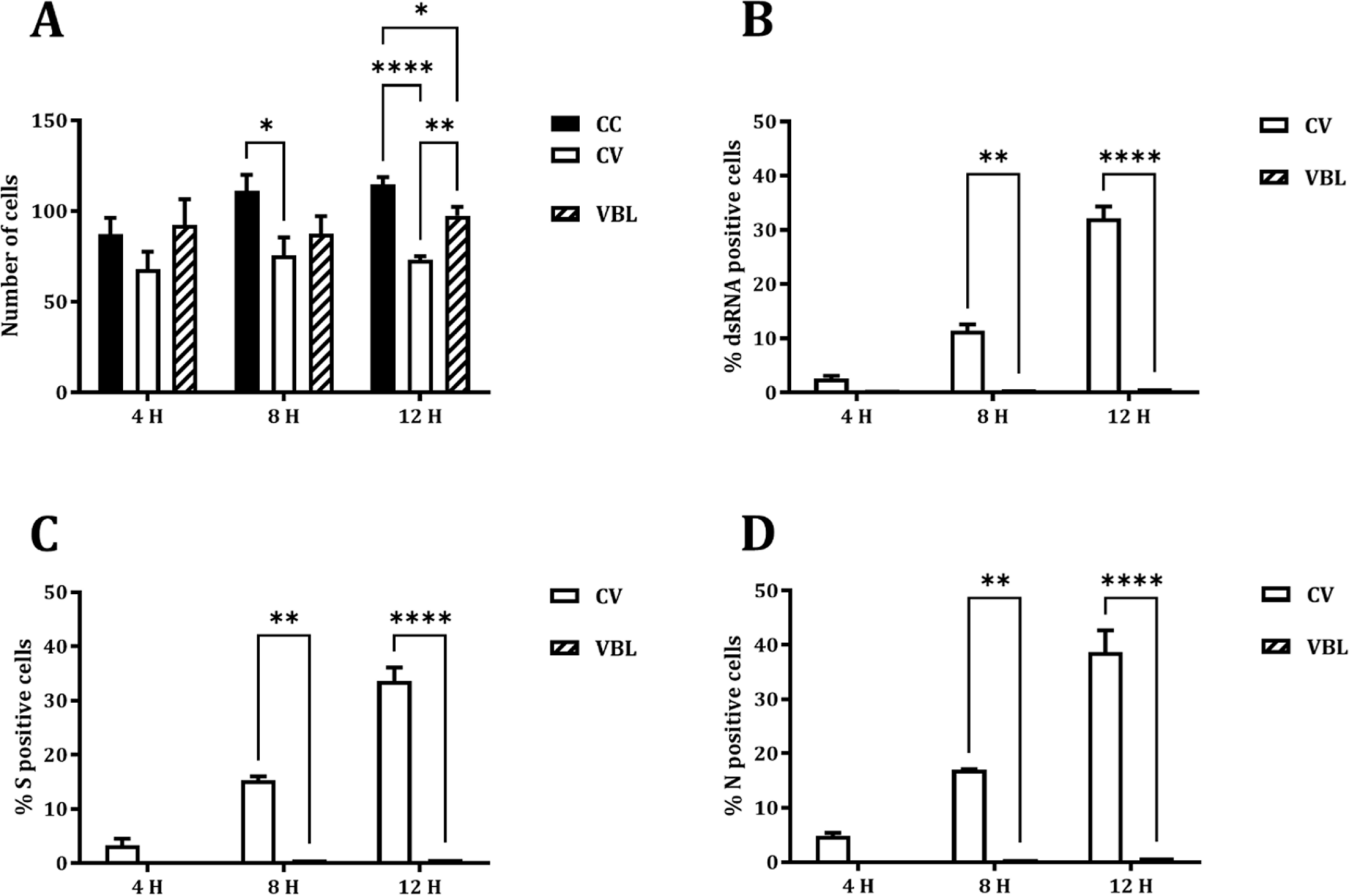
Effects of VBL exposure on the viral replication and protein expression of SARS-CoV-2. (**A**) VERO E6 cells stained with DAPI. (**B**) Expression of dsRNA. (**C**) Expression of viral S protein. (**D**) Expression of viral N protein. Cells were counted at 40× magnification in five random fields/slide for two replicates. Number of cells was expressed as total number; positive cells were expressed as percentage (%). Comparisons were performed by two-way ANOVA (*P < 0.05; **P < 0.01; ****P < 0.0001). CC, cell control; CV, virus control; VBL, VBL-exposed.

To investigate the impact of VBL on different steps of viral replication, the number of viable cells and the amount and localization of viral dsRNA (indicating initiation of viral RNA transcription) and S and N proteins were assessed following VBL exposure. Experiments were run after a 90′ VBL exposure while the virus control culture was sampled at 4, 8, and 12 h post-infection. As reported in Fig. 1, VBL exposure dramatically and comparably decreased the number of dsRNA-, S-, and N-positive cells, recovering the number of viable cells (Fig. 1A).

**Fig 2:**
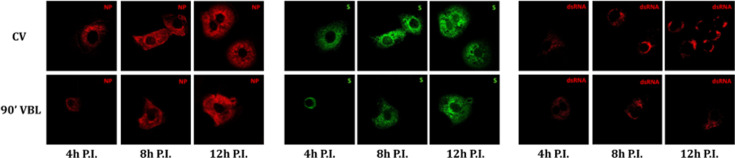
Intracellular localization of SARS-CoV-2 dsRNA and proteins. VERO E6 cells were infected with SARS-CoV-2 (CV) and SARS-CoV-2 after 90′ VBL exposure. Cells were stained at different time points (4, 8, and 12 h post-infection, P.I.) with NP-, S-, and dsRNA-antibody and examined by confocal microscopy.

**Fig 3:**
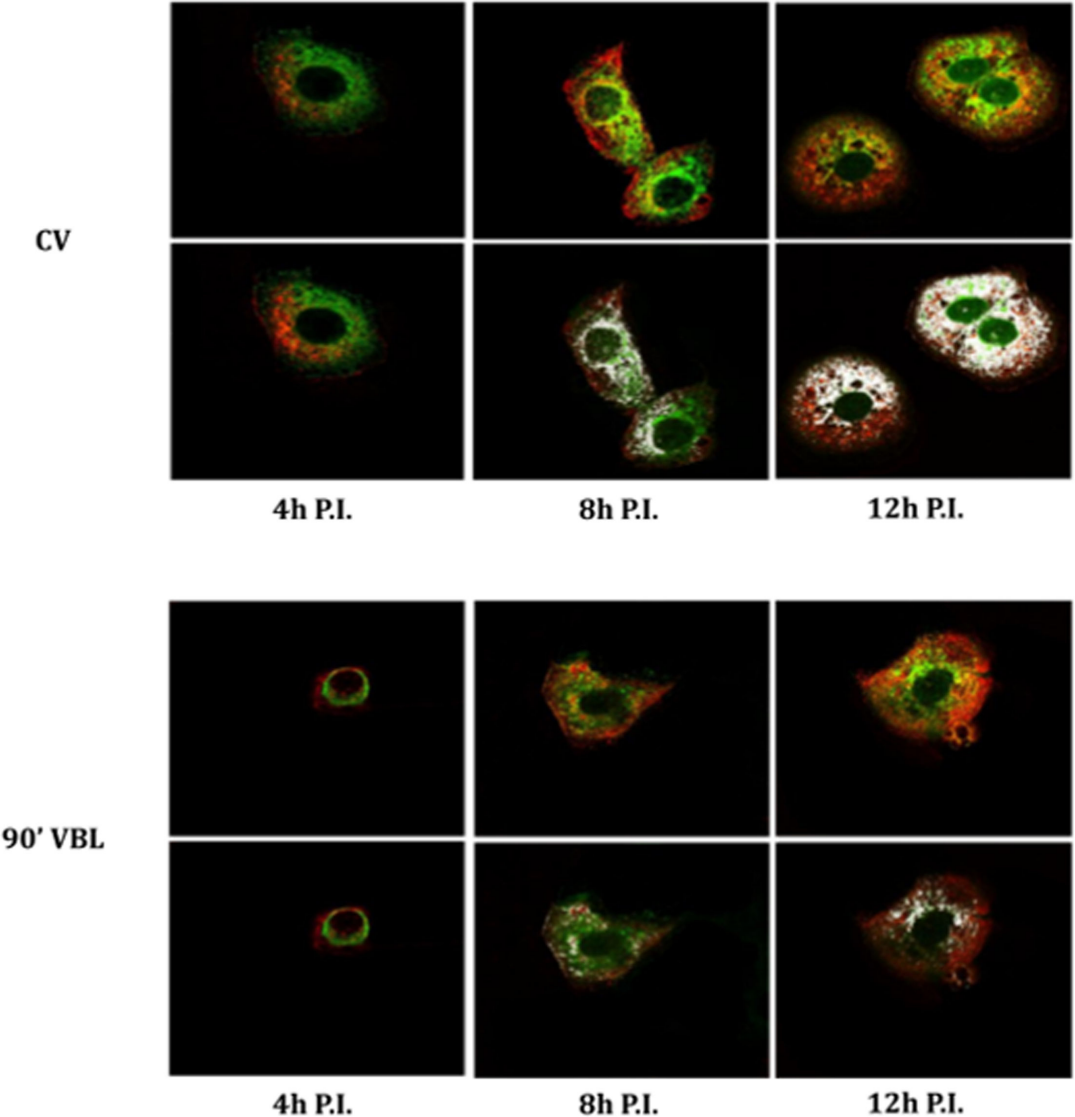
Co-localization of SARS-CoV-2 S and N proteins. VERO E6 cells were infected with SARS-CoV-2 (CV) and SARS-CoV-2 after 90′ VBL exposure. Cells were stained at different time points (4, 8, and 12 h post-infection, P.I.) with NP- and S-antibody and examined by confocal microscopy. Merged signals (upper panels of each condition) and co-localized points (lower panels of each condition) are shown. Co-localized points appear white.

The time-course localization of dsRNA and viral proteins was studied through confocal microscopy. In virus control cultures, dsRNA is expressed from 4 h post-infection, increasing at 8 and 12 h post-infection, and localizes in peri-nuclear foci. N protein is expressed from 4 h post-infection and increases at 8 h post-infection, involving the whole cytosolic compartment. At 12 h post-infection, it moves from the cytosolic compartment toward the cell membrane. S protein is expressed at 4 h post-infection in a perinuclear location and increases at 8 and 12 h post-infection involving the whole cytosolic compartment (Fig. 2). Moreover, the co-localization of N and S starts at 8 h after infection and increases after 12 h (Fig. 3). After 90′ VBL exposure, dsRNA and viral protein expression was greatly reduced; however, similar localization patterns as for virus control were observed (Fig. 2), suggesting that VBL may globally impact cell infection but does not alter the expression and localization of the viral components.

### SARS-CoV-2 RNA quantitative and qualitative analysis

Real-time PCR quantification (qRT-PCR) detected comparable levels of SARS-CoV-2 RNA in the RNA extracts obtained from the 1:3 diluted 90′ VBL-exposed and from the control virus (CV) supernatant (SN) (16.9 and 15.5 Ct, respectively). Likewise, quantification of the extracted RNA with Qubit assay showed a comparable concentration of the VBL-treated and CV SNs (0.52 and 0.45 ng/µL, respectively).

Next-generation sequencing (NGS) analysis of SARS-CoV-2 RNA was performed on 90′ VBL exposure and CV SNs at two time points (0 and 12 h post-infection). The sequences were compared with the reference vaccine strain (NCBI reference sequence: NC_045512). Neither amino acid nor nucleotide differences were observed between the VBL-exposed and CV SNs (Table 2).

**TABLE 2:** Whole-genome NGS analysis of SARS-CoV-2^*a*^

Sample	Pango lineage	Substitutions	aa substitutions
T0 control virus	B.1	C241T-C3037T-C14408T-A23403G-T23569C	ORF1b:P314L-S:0614G
T0 virus after 90′ VBL exposure	B.1	C241T-C3037T-C14408T-A23403G-T23569C	ORF1b:P314L-S:D614G
12 h P.I. control virus	B.1	C241T-C3037T-C14408T-A23403G-T23569C	ORF1b:P314L-S:D614G
12 h P.I. virus after 90′ VBL exposure	B.1	C241T-C3037T-C14408T-A23403G-T23569C	ORF1b:P314L-S:0614G

^*a*^ The samples belong to the Pango B.1 genomic lineage and share the same genomic mutations: C241T, C3037T, C14408T, A23403G, and T23569C with respect to the reference ancestral virus. These mutations correspond to identical amino acid substitutions in each sample, namely ORF1b:P314L and S:D614G. The analysis suggests that exposure to VBL under the experimental conditions described in the text did not introduce any change in the virus genome.

### TCID_50_/mL reduction of SARS-CoV-2 after VBL exposure with different medium dilutions

**Fig 4:**
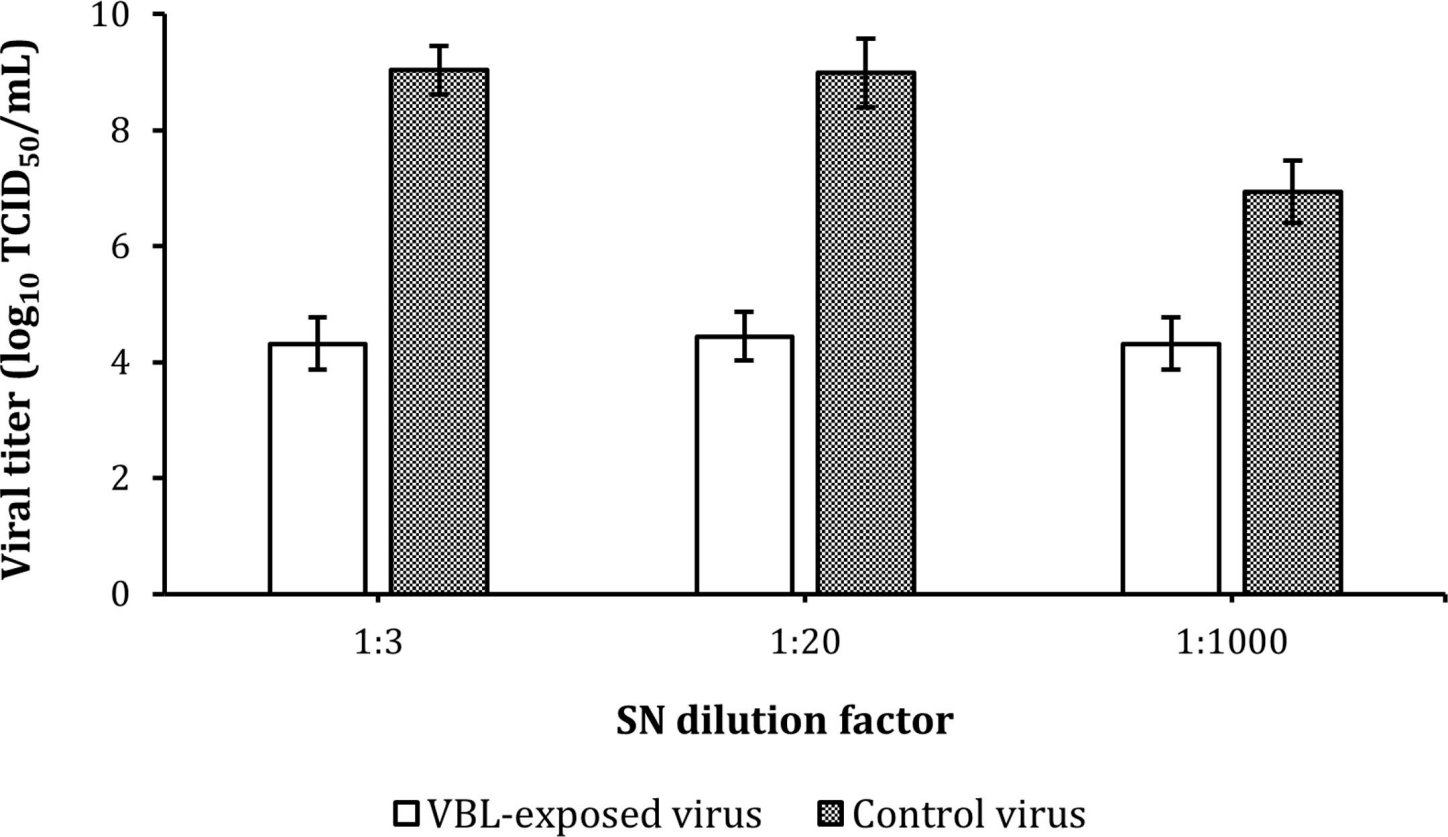
Viral load reduction of SARS-CoV-2 in different dilutions of culture medium exposed to VBL for 90′. The viral SN was diluted in PBS at different concentrations to reduce the presence of DMEM culture medium during VBL exposure. The results obtained showed a TCID_50_/mL logarithmic reduction of the exposed samples with respect to CVs of 4.719 log_10_ (for the SN dilution factor of 1:3), 4.543 log_10_ (for the SN dilution factor of 1:20), and 2.620 log_10_ (for the SN dilution factor of 1:1,000); percentage reductions achieved were 99.998%, 99.997%, and 99.76%, respectively. The dilution of the medium certainly affected virus survival, suggesting that its presence plays a secondary but relevant role in VBL-mediated viral inactivation. Results were expressed as the logarithm of TCID_50_/mL and relative 95% confidence interval (CI) obtained using the improved Kärber.

Exposure of the viral SNs to VBL showed a remarkable reduction of infectious titer at all three different conditions tested (diluted 1:3, 1:20 and 1:1,000 in phosphate-buffered saline [PBS]), compared to the CV (Fig. 4). However, residual virus titer following VBL exposure was comparable in the three conditions, and the difference in fold-change reduction appeared to be driven by the decrease in untreated virus titer due to the different dilution factors. Specifically, TCID_50_/mL without and with exposure to VBL decreased from 9.03 log_10_ (CI: 8.61–9.45) to 4.31 log_10_ (CI: 3.86–4.77) with 1:3 diluted SN; from 8.98 log_10_ (CI: 8.3–9.5) to 4.44 log_10_ (CI: 4.02–4.86) with 1:20 diluted SN; and from 6.93 log_10_ (CI: 6.39–7.48) to 4.31 log_10_ (CI: 3.86–4.77) with 1:1,000 diluted SN (Fig. 3). The table with extended data on viral titer and fold change values is included in the Supplementary Information (Table S1).

### VBL treatment of SARS-CoV-2 with antioxidants

Antioxidants were added to the virus under the same experimental conditions as above to study the effect of oxidative stress induced by VBL exposure. The antioxidants tested included (i) 5 and 0.5 mM N-acetylcysteine (NAC), (ii) 5 and 0.5 mM ascorbic acid (AsA), and (iii) 0.03 and 0.003 mM superoxide dismutase (SOD).

**Fig 5:**
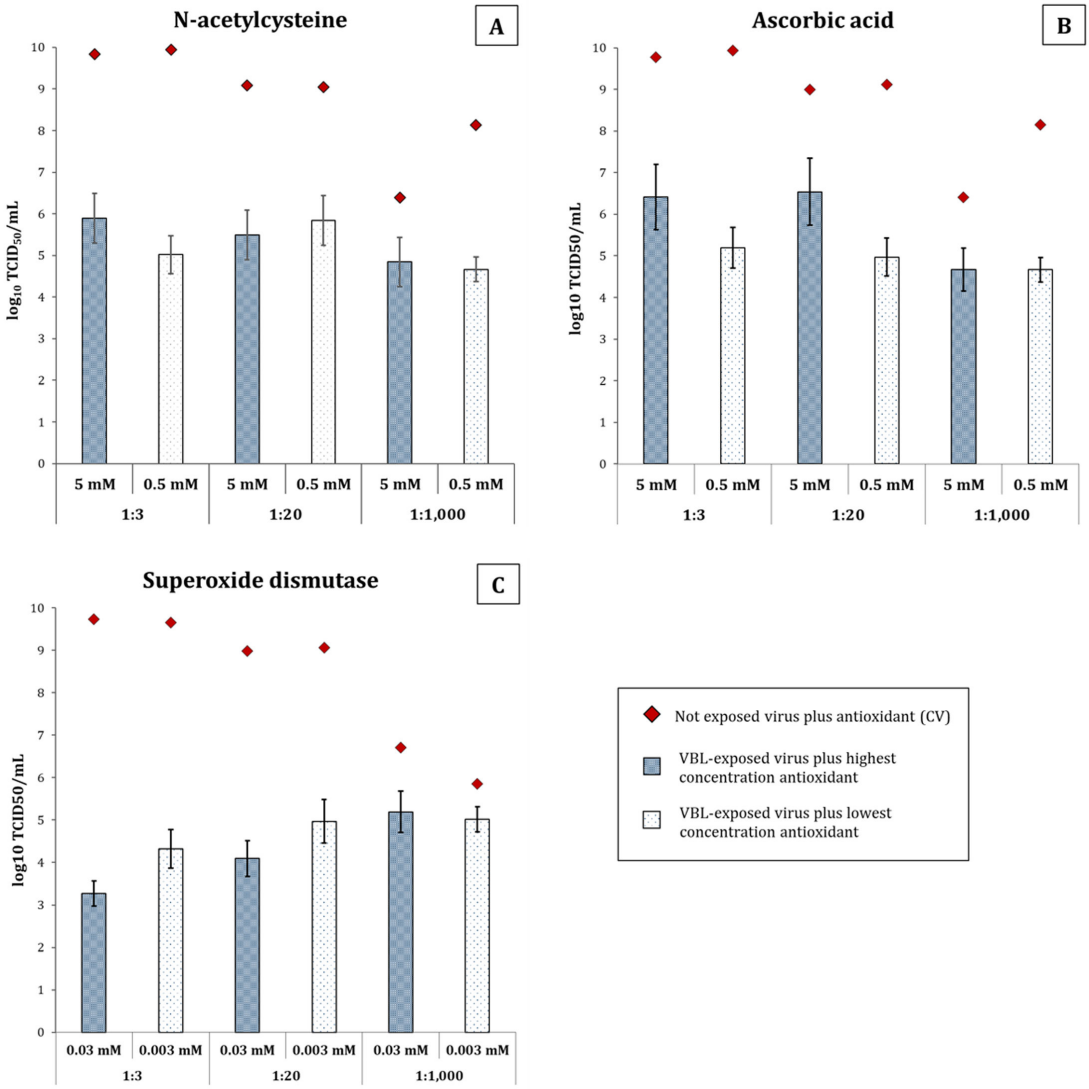
Evaluation of antioxidant protection against VBL-induced inactivation of SARS-CoV-2 in different dilutions of culture medium. The viability of SARS-CoV-2, as measured by log_10_ TCID_50_/mL, is depicted in this figure after a 90-min exposure to VBL. The virus was incubated in DMEM diluted 1:3, 1:20, or 1:1,000 in PBS during irradiation, and various antioxidants were added at two concentrations. The goal was to ascertain whether these antioxidants could shield the virus from the oxidative stress caused by VBL. In the panels, the results are presented for (**A**) NAC at 5 mM (blue patterned bars) and 0.5 mM (white patterned bars), (**B**) AsA at 5 and 0.5 mM, and (**C**) SOD at 0.03 and 0.003 mM. The whiskers of the bars represent the 95% confidence interval. The titers of the non-exposed control virus (CV) are represented by red diamonds. The CV was incubated under identical conditions (medium dilution, antioxidant type, concentration, and time) without VBL exposure, and they served as a baseline for maximum viability. The titers of the CV range from 8 to 10 log_10_ TCID_50_/mL, according to the specific condition.

As shown in Fig. 5 and Table S2, the higher concentrations of AsA and NAC at the lower SN dilutions (1:3 and 1:20) resulted in higher virus survival after VBL exposure. Specifically, at the 1:3 SN dilution, the reduction of VBL-treated virus decreased from 4.71 log_10_ in the absence of antioxidants to 3.94 log_10_ and 3.36 log_10_ with the addition of 5 mM NAC and of 5 mM AsA, respectively. As for the 1:20 diluted SN, the reduction ranged from 4.54 log_10_ in the absence of antioxidants to 3.59 log_10_ and 2.46 log_10_ with the addition of 5 mM NAC and of 5 mM AsA, respectively. Even at the lower concentrations (0.5 mM), both NAC and AsA slightly increased virus survival with the 1:20 diluted SN, allowing a variation of −8.81% (4.15 log_10_) and −29.51% (3.20 log_10_), respectively, compared to the VBL-treated SNs without antioxidants (4.54 log_10_). SOD proved to be almost ineffective in increasing SARS-CoV-2 survival at the lower SNs dilutions (1:3 and 1:20), regardless of its concentration. In contrast, at the highest SN dilution (1:1,000), SOD allowed a higher rate of virus survival than NAC and AsA. Specifically, the titer of the VBL-treated virus decreased from 2.62 log_10_ in the absence of antioxidants to 1.55 log_10_ with the addition of 5 mM NAC, 1.73 log_10_ with the addition of 5 mM AsA, and 1.51 log_10_ with the addition of 0.03 mM SOD, with a variation of −40.79%, −33.72%, and −42.16%, respectively. The increased viral survival rate disappeared at the lowest concentration of antioxidants, except for SOD. Indeed, with 0.003 mM SOD, the reduction ranged from 2.62 log_10_ to 0.83 log_10_.

Overall, the addition of AsA (and to some extent NAC) appears to be more protective at the 1:3 and 1:20 dilutions of the virus, suggesting that the presence of residual DMEM medium and the photosensitizers that it contains may in part drive VBL-mediated oxidation of the virus.

### Protein carbonylation assay

**Fig 6:**
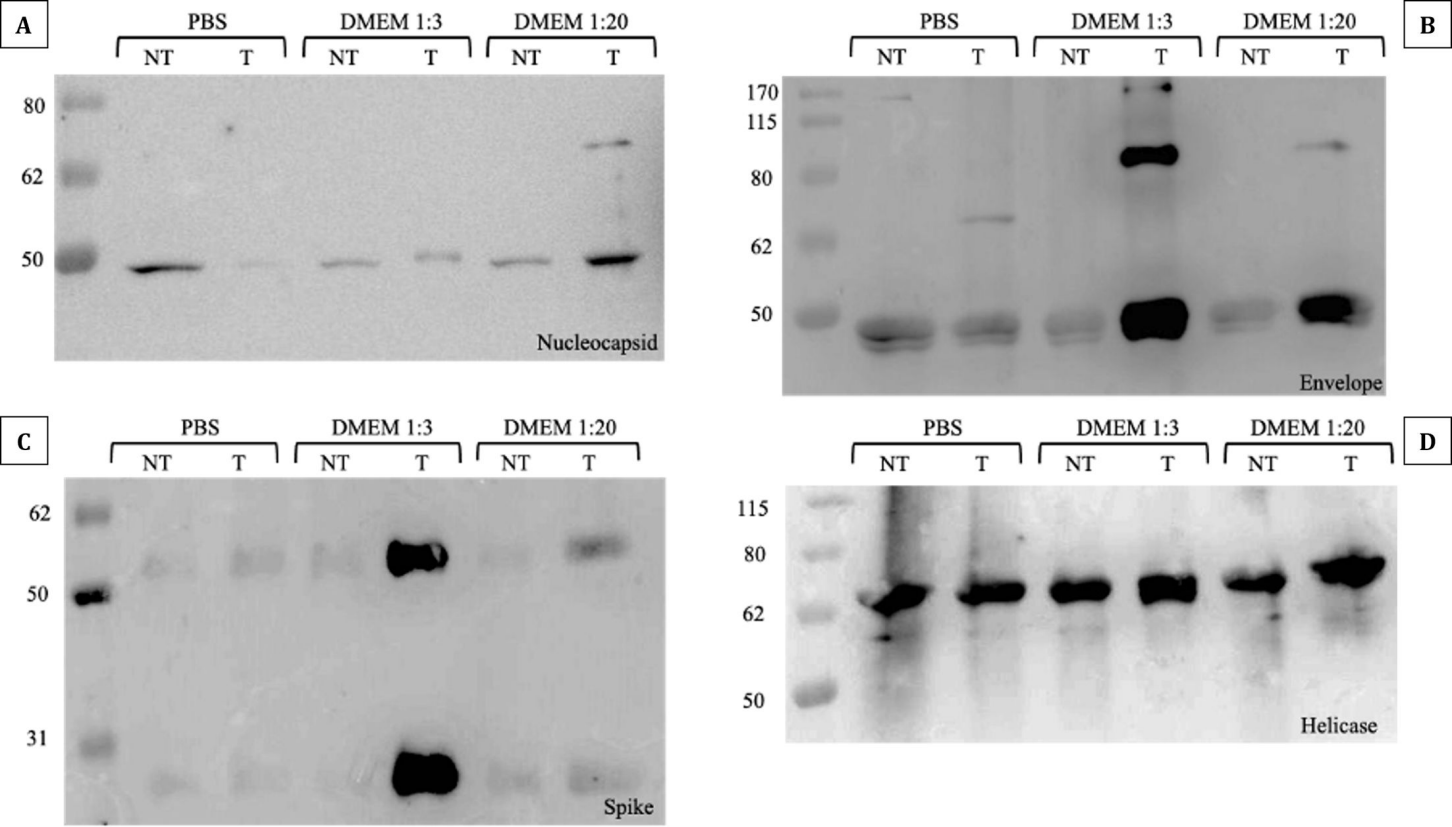
Protein carbonylation assay results. Detection of carbonylated proteins derivatized with DNPH and analyzed by Western blot in the presence (T) or absence (NT) of VBL exposure. (**A**) Protein N exposed to VBL showed no visible signs of carbonylation compared to unexposed controls and different incubation conditions with different dilutions of the culture medium. In general, slight protein oxidation seems to be visible in all samples, probably due to the experimental conditions under which the tests were carried out. (**B**) Protein E showed signs of carbonylation in samples exposed to VBL and incubated with DMEM medium dilutions of 1:3 and 1:20. In this case, the presence of higher medium concentrations seems to have affected the oxidative state of the protein. Again, a slight presence of protein oxidation is visible in all samples, probably due to the experimental conditions of the assay. (**C**) Protein S showed significant carbonylation in the 1:3 dilution of medium exposed to VBL, although a slight level of carbonylation is also present in the sample incubated with the 1:20 dilution. Again, it appears that the concentration of medium increases the degree of protein carbonylation in the presence of VBL. (**D**) Protein NSP13 showed signs of extensive carbonylation regardless of the variables examined. Again, the results could be due to a combination of factors related to the intrinsic properties of the protein and the experimental conditions under which the tests were carried out. Nevertheless, no differences were found between the samples exposed and those not exposed to VBL.

SDS-PAGE and Western blotting experiments on N, S, E, and helicase (NSP13) proteins were conducted to determine whether VBL irradiation reduces viral replication by altering viral protein structure. As shown in Fig. 6, the S and E proteins exhibited higher oxidation levels compared to the untreated control when irradiated in the presence of culture medium. Specifically, oxidation was more pronounced at the 1:3 dilution compared with the 1:20 dilution, while both conditions differed markedly from the PBS control. For the N protein, the 1:20 dilution showed a more intense band compared with the control, along with the appearance of a fainter, higher-molecular-weight band, whose intensity was comparable to that observed in the control at the expected molecular weight. Additionally, at the 1:3 dilution, the N protein band showed a slight molecular weight shift in the VBL-exposed sample. No differences were observed for the NSP13 protein across the experimental conditions tested. These results suggest that oxidative modifications following VBL exposure primarily affect surface proteins.

## DISCUSSION

The sensitivity of viruses to photodynamic inactivation was first reported in the 1930s (37). However, over the last 40 years, photodynamic procedures for virus inactivation have garnered increasing attention (38). This increased interest stems from the development of new active photosensitive molecules, advancements in light technologies (lasers, LEDs, and portability) (39), and the recent pandemic emergency.

This study investigated the mechanisms underlying SARS-CoV-2 inactivation in response to VBL exposure. The results are varied and heterogeneous, indicating the inactivation process is likely influenced by multiple interconnected mechanisms rather than a single cause.

### Cell cycle regulation and RNA integrity

Initial investigation focused on potential light interference with the viral replication cycle and possible viral RNA damage.

Experimental data showed that VBL exposure does not cause significant changes in the viral replication cycle, suggesting the replication process remains largely unaffected. Indeed, analysis of the time course and cellular localization of viral components indicated the main difference between VBL-exposed and non-exposed viruses lies primarily in the concentration of viral particles infecting the cells. These results, combined with the TCID_50_/mL data, led us to hypothesize that light-induced virus inactivation occurs before viral entry into the target cell. Other studies support this hypothesis, suggesting irreversible damage to viral structures occurs before host cell entry (31, 40).

Similarly, results on genetic material integrity showed that damage to ssRNA does not directly affect viral inactivation. Both qRT-PCR and NGS revealed no significant alteration of viral RNA. These findings exclude nucleic acid degradation as the primary mechanism and instead highlight other potential cellular and viral structural perturbations. Further studies support this, ruling out direct VBL-induced damage to the viral genome that could irreversibly affect the replication cycle (31, 41). Furthermore, we found no evidence that other viral components, such as structural and non-structural proteins involved in transporting and protecting genetic material within the host cell (e.g., N protein) (42), are the primary target of VBL.

### Indirect assessment of oxidative stress

Results from the previous experiments allowed us to exclude, with reasonable confidence, some traditionally considered factors in virus inactivation, shifting the focus to remaining vital components like proteins and the cell membrane. Given the properties of the wavelength used (405 nm), an additional investigation step was necessary to identify the possible type of damage caused by VBL. Two potential mechanisms emerged: (i) oxidative damage, where photosensitive elements produce ROS, triggering a cascade primarily damaging the membrane and proteins (43), and/or (ii) conformational changes in key viral structural proteins (44), potentially compromising virus adhesion and cellular uptake, thus preventing replication.

Experiments incubating the virus with antioxidants (initially only in 1× PBS) during VBL exposure tested the first hypothesis, revealing that oxidative damage significantly contributes to VBL-induced virus inactivation. Indeed, treatment with NAC, AsA, and SOD at different concentrations partially but significantly prevented SARS-CoV-2 inactivation (reducing it from 99.99% without antioxidants to 99.96% with NAC 0.5 mM and 85.43% with SOD 0.003 mM) (Fig. 5). These data highlight the role of ROS produced during VBL exposure as mediators of oxidative stress, indicating their interaction with viral components potentially affects structural stability and functionality (39).

Based on the hypothesis that oxidative stress is a key inactivation mechanism, we evaluated DMEM cell culture medium as a potential primary source of ROS production. Several studies suggest photosensitive molecules like riboflavin in suspension media may act as primary ROS sources upon VBL exposure, potentially compromising viral viability before cell culture incubation (31, 40). In this context, our results seem to partially diminish the role of DMEM medium as the primary source of ROS production. The same reduction (>99.99%) was achieved when the virus was exposed to VBL at 1:3 and 1:20 dilutions in PBS. Therefore, there appears to be no significant difference in virus viability regardless of the culture medium concentration used. However, exposing the virus to VBL at a 1:1,000 dilution in PBS yielded 99.76% inactivation. While this slight difference seems biologically insignificant, it does not allow us to completely exclude the suspension medium’s role as an adjunct in the inactivation process, suggesting it may play a secondary rather than a central role. Several studies appear to confirm this hypothesis (32, 33, 45), and our IAV and IBV inactivation results also seem to corroborate it. Indeed, although the suspension media differed (UltraMDCK for IAV/IBV vs DMEM for SARS-CoV-2), photosensitive components should also be present and contribute to influenza virus inactivation, yielding similar results to those seen with SARS-CoV-2. However, this was not observed, suggesting the inactivation process is likely driven by intrinsic features of the virus morphology. This remains speculative, as comparing photosensitive element concentrations between DMEM and the proprietary UltraMDCK medium is not possible. Subsequent experiments, exposing the virus to different DMEM dilutions and antioxidant concentrations, partially confirmed the medium’s secondary role in inactivation, showing a minimal but measurable increase in cell viability correlating with its concentration. This increase was proportional to both medium and antioxidant concentrations, with AsA generally showing better a ability to reduce oxidative stress. Survival increased to a maximum of 0.5% with 5 mM AsA in DMEM 1:20. Although minimal, this increase may offer a partial estimate of the medium’s effect, but additional tests are required for accurate measurements with high confidence.

### Protein oxidation and structural integrity

Experiments were conducted on three structural (S, N, and E) and one non-structural (NSP13) SARS-CoV-2 protein to test the hypothesis that oxidative stress could be induced by light-sensitive endogenous or exogenous molecules. Proteins were incubated in neutral medium (1× PBS) and different DMEM dilutions (matching cell viability tests) to evaluate the direct effect of light. The aim was to confirm literature findings suggesting significant culture medium involvement in S protein (and partial N protein) oxidation (31, 34, 46, 47), or to partially validate our previous results with DMEM. The hypothesis of VBL-induced conformational change or protein damage was not completely rejected; however, confirming it depended on detecting protein degradation via SDS-PAGE and Western blotting, which was not observed.

Exposing the proteins to VBL in 1× PBS showed no significant oxidative stress-induced changes or molecular weight alterations. This ruled out the possibility that the protein alone promotes viral inactivation. Supporting this, oxidative damage to viral proteins was most evident in tests using DMEM as the incubation medium (particularly with S and E proteins), suggesting DMEM could act as a secondary ROS producer in the presence of VBL. This suggests oxidative stress might affect the structural and functional integrity of these proteins, partially confirming literature reports (39, 48). The S protein, critical for SARS-CoV-2 entry and interaction with the host cell ACE2 receptor (49), showed oxidative changes that could affect its binding affinity or conformational stability. Such structural changes could hinder viral infection, as damaged S proteins might be unable to perform their cellular entry function, primarily via the endosomal pathway (50), consistent with entry dynamics observed in VERO E6 cells (51).

Indeed, oxidative processes can induce post-translational modifications altering protein structure, folding, net charge, and hydrophobic/hydrophilic balance (52). However, this assay cannot determine whether these oxidative modifications led to conformational changes affecting protein interaction or stability, and thus potentially impacting viral infectivity. Furthermore, the relationship between function loss and protein oxidative damage is not direct. Nitration and carbonylation can negatively impact target proteins, but under stress conditions, these modifications might even be beneficial (53). An oxidized protein might retain normal or only slightly reduced function (54), without affecting cellular structure or viral replication. This latter point is strongly supported by the cell viability results, leading to the hypothesis that: (i) proteins most likely do not undergo direct VBL-induced alterations; (ii) the suspension medium has a secondary effect, contributing to inactivation likely via membrane protein oxidation; but (iii) medium-induced protein oxidation alone cannot explain the inactivation results obtained, even at the 1:1,000 DMEM dilution.

### SARS-CoV-2 membrane composition and phospholipid oxidation

While protein oxidation alone may not fully explain viral inactivation, combining it with membrane phospholipid oxidation might help clarify the complex phenomenon observed, assigning a secondary but supportive role to protein oxidative damage in reducing virus survival.

Lipid peroxidation, mainly ROS-driven, is well-studied and known to adversely affect membrane integrity by altering lipid bilayer fluidity and permeability. The lack of oxidative damage control mechanisms exposes viral envelope phospholipids to oxidative changes (55). Free radicals such as AAPH (2,2′-Azobis(2-amidinopropane) dihydrochloride) (56) and DBHN (di-tert-butyl hyponitrite) (57) can accelerate oxidation, increasing the structural instability of viral phospholipids. VBL-based inactivation could target this viral membrane vulnerability, as lipid peroxidation can compromise the viral envelope, rendering it non-functional and limiting its ability to infect host cells.

This assumption seems supported by two key previous findings: (i) the different survival rates of IAV/IBV vs. SARS-CoV-2 after VBL exposure, and (ii) the higher SARS-CoV-2 survival when co-incubated with SOD during VBL exposure.

Regarding the first finding, differences in the lipid bilayer composition of the two virus types could account for their varying susceptibility to oxidative stress and VBL exposure. Indeed, according to a recent study by Saud et al. (58), the SARS-CoV-2 lipid bilayer is composed mainly of phosphatidylcholine), phosphatidylethanolamine (PE), and phosphatidylinositol (PI), with modest amounts of phosphatidylserine (PS), phosphatidylglycerol (PG), and sphingolipids (SLs) like ceramide and dihydroceramide (DHCer). Conversely, cholesterol, triacylglycerols, free cholesterol, and sphingomyelin (SM) appear largely absent (58). These latter components, more resistant to oxidative stress than glycerophospholipids, are essential for maintaining membrane fluidity and flexibility and provide additional support against prolonged stress (55). The influenza virus membrane presents a different composition. It is composed mainly of cholesterol (up to 40%) (59), SLs such as SM, glucosyl/galactosylceramide, and DHCer, phospholipids (PL) including PS and PE, and small amounts of PG (60). The virus’s complex homeostatic equilibrium may depend on this intrinsic variability in membrane composition, where type I or II photoinitiators (e.g., Fe(III) atoms, O•−, hydroxyl radicals, ozone, and H_2_O_2_) can trigger widespread, irreversible lipid peroxidation (61), induced by stressors like VBL.

For the second finding, lipid peroxidation could explain SOD’s superior performance compared to the other antioxidants. In oxygenated solutions, ionizing radiation generates superoxide, while in deoxygenated water, it produces hydroxyl radicals and solvated electrons triggering lipid autoxidation cascades. At low concentrations, SOD neutralizes superoxide radicals, acting as a control mechanism for oxidative stress in complex systems like eukaryotic cells (55). In our study, the most protective effects were seen when SOD was incubated and exposed in the 1:1,000 supernatant dilution in PBS. When incubated with the virus and DMEM, the results were negligible (regardless of concentration), likely due to the secondary ROS generation process caused by DMEM.

Further studies using advanced techniques are necessary to definitively assess the preservation state of the viral membrane after VBL exposure and confirm its precise role in the virus inactivation mechanism.

### Conclusion

VBL inhibits SARS-CoV-2 and influenza virus replication primarily by inducing oxidative stress to the viral membrane, with an emphasis on phospholipids and, to a lesser degree, viral proteins. The results obtained allow us to hypothesise that the oxidative degradation of phospholipids and the reduced presence of cholesterol and sphingolipids can alter the viral membrane, reducing the ability of the virus to maintain its structural integrity or interact with host cells. The results of this study underscore the potential of VBL as a non-invasive disinfection tool for inactivating viruses such as SARS-CoV-2. This method may be particularly useful in situations when direct viral exposure is unavoidable, such as in healthcare facilities or air filtering systems.

In conclusion, VBL has great potential as an innovative, affordable, and scalable disinfection tool, including against viruses. However, further studies are needed to optimize its use, particularly in settings where safety and efficacy must be ensured. Integrating antioxidant therapies with VBL could be a more effective viral inactivation strategy than using VBL alone, thus improving therapeutic applications and environmental disinfection. However, it is essential to recognize some limitations of the study, including the limited number of viruses tested and the need to consider the complexity of real-world settings. In this regard, *in vivo* studies might be useful to better assess prospects. The potential of VBL as an environmentally friendly and safe alternative to traditional disinfection methods is considerable, but further research is needed to define the optimal application parameters, such as the amount of energy, duration, source distance, and exposure time that could be most effective to achieve viral inactivation.

## MATERIALS AND METHODS

### Experimental setting and photoradiometric simulation

**Fig 7:**
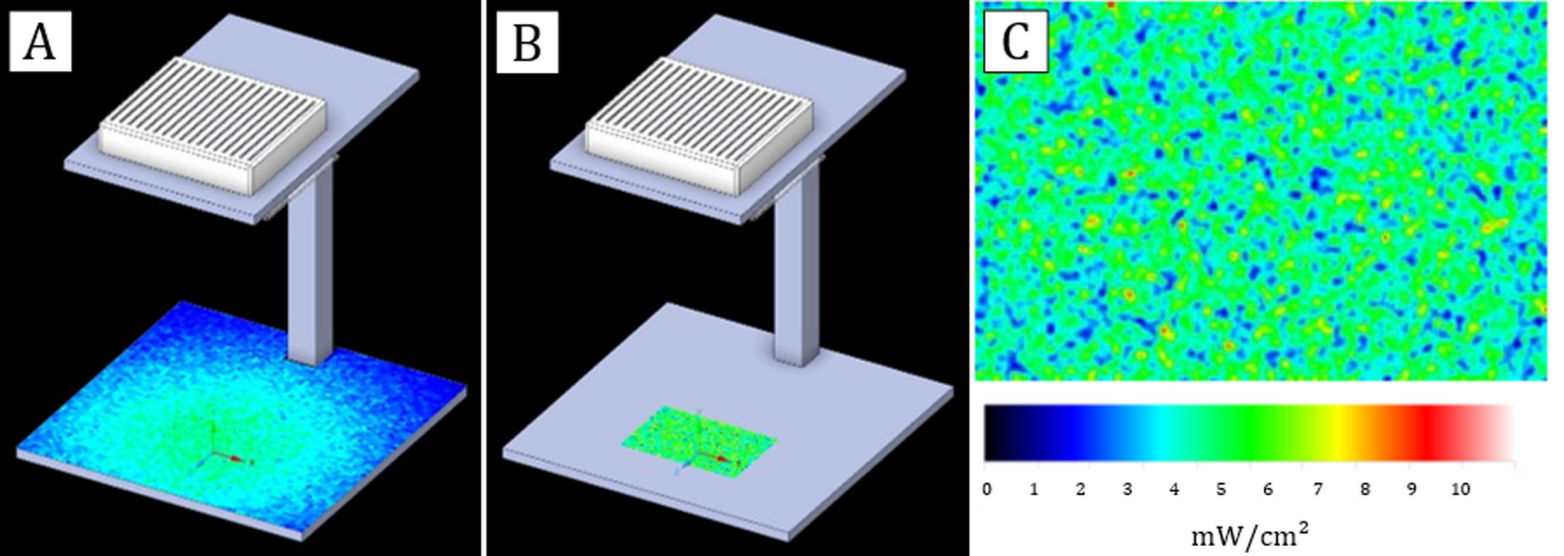
3D representation of the light source and VBL light distribution. (**A**) Light irradiance simulation on the plane realised with Ansys Speos software. The radio-photometric simulation was carried out considering the power of each light source (the nine LEDs at 405 nm), the distance of the light sources from the plane, and empirical measurements taken at various points on the plane using the Avantes ULS2048CL EVO spectrophotometer. As expected, the highest irradiance (section in green) was measured on the perpendicular plane section below the source (4.3 mW/cm^2^), while an average irradiance decrease of 83% (0.7 mW/cm^2^) was recorded on the lateral plane sections (section in blue). (**B**) Irradiance on the multi-well plate. The marked area on the plane (in green) represents the point, where the multi-well plate is positioned during the virus and protein exposure experiments. Before starting the experiments, the temperature at the point of maximum light exposure was measured to exclude it as a possible risk factor. The measured temperature was not different from room temperature (23°C, 50% relative humidity). (**C**) Detail of the light distribution on the plate.

The VBL prototype lamp consisted of nine LEDs centred at 405 nm (±4 nm) positioned on a square metal stand. The light source was supported by a metal scaffold attached to a 30 cm × 30 cm square base at 32 cm from the light sources, on which the profile of a multi-well plate was outlined to perform the tests correctly (Fig. 7A).

The characterization of the electromagnetic spectrum was performed using an Avantes ULS2048CL EVO spectrophotometer (Avantes, Apeldoorn, the Netherlands). The energy map of the multi-well plate was measured to obtain the distribution of light irradiance (Fig. 7B). The average irradiance measured within the wells of the plate was 4 mW/cm^2^.

The test setup, the number of LEDs, and their position in the device were designed using Ansys Speos software. Subsequently, a simulation was conducted to ensure that the light distribution was uniform in all wells of the plate, and then empirically confirmed the simulated values by measuring the light distribution at 405 nm (Fig. 7C). The following photometric simulation parameters were set:

- radiant flux of VBL LEDs = 1.4 W, taken from the LED datasheet as the median value of the intermediate production bin;
- Lambertian type of intensity with an aperture angle of 130°; and
- 7% absorption of VBL by the polymer cover protecting the LEDs.

Fig. S1 shows the specifications of the type of LEDs used and the characterization of their light spectrum, carried out using the Avasoft measurement software (Avantes, Apeldoorn, the Netherlands).

### SARS-CoV-2 and influenza viruses

The SARS-CoV-2 variant used for the experiments was the wild-type B.1 (GISAID code EPI_ISL_2472896), kindly provided by the Department of Biomedical and Clinical Sciences, Luigi Sacco, University of Milan, Italy. The cell line used to propagate the viral stock was VERO E6, a cell line with epithelial morphology originally isolated from the kidney of an African green monkey (ATCC CRL-1586). VERO E6 cells were propagated in DMEM high glucose medium (Euroclone, Pero, Italy) supplemented with 10% fetal bovine serum (FBS; Euroclone, Pero, Italy) and 1% streptomycin/penicillin (PS) (Euroclone, Pero, Italy) in a humidified incubator at 37°C with 5% CO_2_. After propagation, the viral stock was titrated in VERO E6 as previously described (62). To perform viral infection, the same DMEM medium but with 1% FBS concentration was used.

The influenza viruses used for the experiments were A/Wisconsin/588/2019 (H1N1), as representative of IAVs, and B/Austria/1359417/2021 (B/Victoria lineage) (NIBSC 21/218), representative of IBVs. The cell line used to propagate both viruses was the Madin-Darby Canine Kidney (MDCK) (ATCC CCL-34). MDCK cells were cultured in Eagle’s minimum essential medium (Lonza, Milano, Italy) supplemented with 2 mM l-Glutamine (Lonza, Milano, Italy), 1% non-essential amino acid solution (Sigma-Aldrich, St. Louis, MO, USA), 100 units/mL PS mixture (Lonza, Milano, Italy) and 10% FBS (Euroclone, Pero, Italy), in a humidified incubator at 37°C with 5% CO_2_. To perform viral infection, UltraMDCK serum-free medium (Lonza, Milano, Italy) with 1% PS and 1 µg/mL Trypsin TPCK treated (Sigma-Aldrich, St. Louis, MO, USA) was used. After propagation, the viral stock was titrated in MDCK cells as previously described (63).

### Virus exposure to VBL

Viral suspensions of SARS-CoV-2 and influenza (IAV and IBV) were exposed to VBL, in DMEM or UltraMCDK medium in microtiter wells (300 µL per well) of a multi-well plate, at different doses of light energy: 22′30″ (dose of 5.4 J/cm^2^), 45′ (10.8 J/cm^2^), and 90′ (21.6 J/cm^2^). In all exposures, a non-VBL-exposed virus was used as control.

In all subsequent experiments, the SARS-CoV-2 SN was exposed for 90′ of continuous irradiation under the VBL lamp at a 30 cm distance from the light source, with an irradiation of 4 mW/cm^2^. Each virus SN was tested at 1:3, 1:20, and 1:1,000 dilution in PBS to account for the cell culture medium effect. The same experiments were performed with the addition of alternative antioxidants: NAC (5 and 0.5 mM) (VWR International, Radnor, PA, USA), AsA (5 and 0.5 mM) (VWR International, Radnor, PA, USA), and SOD (0.03 and 0.003 mM) (Thermo Fisher Scientific, Waltham, MA, USA). In all experiments, non-VBL-exposed SARS-CoV-2 (without the addition of antioxidants) was used as control.

### Immunofluorescence assay

The spatiotemporal distribution of the SARS-CoV-2 N protein, S protein, and dsRNA was investigated by immunofluorescence on VERO E6 cells infected with the VBL-exposed and unexposed virus. The cells were seeded 24 h before infection on 24-well plates and covered with sterile borosilicate cover glasses (diameter 13 mm, thickness no. 1.5, VWR International). On the day of infection, the SARS-CoV-2 stock was irradiated under the VBL lamp for 90′ and then used to infect the pre-seeded VERO E6 cells. In parallel, VERO E6 cells were infected with the unexposed CV at 0.2 MOI, and uninfected cells were used as cell control. The infected cells, both with the VBL-exposed virus and with the CV, and the uninfected cells were then fixed with 10% paraformaldehyde for 20′ at 4, 8, and 12 h post-infection.

The immunofluorescence assay was then performed as previously reported (62). Briefly, after the permeabilization step (0.5% Triton X-100, 7′), non-specific binding sites were blocked in 3% bovine serum albumin (BSA; Life Technologies, Carlsbad, CA, USA) for 1 h, then cells were incubated overnight with primary antibody (anti-dsRNA, anti-NP, or anti-S) diluted in PBS containing 0.5% BSA + 0.5% Triton X-100. After washing steps (PBS containing 0.5% BSA + 0.5% Triton X-100 + 0.05% Tween20), the cells were incubated for 1 h with AlexaFluor 488- or 568-labeled secondary antibody (Life Technologies, Carlsbad, CA) diluted in PBS with 0.5% BSA + 0.5% Triton X-100. Coverslips were mounted in Fluoromount Aqueous Mounting Medium (Merck, Darmstadt, Germany) and pictures of stained cells were taken through a confocal microscope (Zeiss LSM500; Carl Zeiss, Germany). The number of viable cells was determined by the count of cells stained with 4′,6-diamidino-2-phenylindole (DAPI; Sigma-Aldrich, St. Louis, MO, USA) and randomly (five fields) counted at 40× original magnification to the confocal microscope. To determine the percentage of SARS-CoV-2 infection, dsRNA-, N-, or S-expressing cells were counted, and data were reported as the percentage of positive cells/slide. Reagents for immunofluorescence (PBS, Triton X-100, and Tween20) and anti-dsRNA, anti-N, and anti-S antibodies were obtained from Merck (Darmstadt, Germany).

The number of cells was determined by the count of cells stained with DAPI and randomly counted (five fields) at 40× original magnification. dsRNA-, S-, and N-expressing cells were counted, and data were reported as the percentage of positive cells/slide. Images were pre-processed using ImageJ software (US National Institutes of Health, Bethesda, MD, USA). The colocalization of S and N proteins was represented as white dots using the colocalization plug-in.

### Virus titration after VBL irradiation

All viral SNs were harvested and titrated to evaluate the rate of virus inactivation and to test whether the SN retained the ability to infect cells. At the end of VBL exposure to SARS-CoV-2, IAV, and IBV, titration was performed as previously reported (62–64).

At the beginning, SARS-CoV-2, IAV, and IBV were titrated in serial 1 log_10_ dilutions to obtain a TCID_50_ on 96-well culture plates of VERO E6 or MDCK cells, respectively. After 72 h of incubation at 37°C with 5% CO_2_, plates were observed for the presence of cytopathic effect (CPE) by means of an inverted optical microscope. The viral titer was calculated using the Reed-Muench method and expressed as TCID_50_/mL.

ELISA assay and quantification of CPE were initially performed as screening experiments to optimize experimental conditions, to identify influencing variables, and to determine the most suitable readout for subsequent analyses. Notably, all three titrations revealed a 3 log_10_ reduction of the VBL-exposed virus titer compared to the untreated control, as we can observe in Table S3. Considering these results, we decided to proceed using the quantification of CPE as readout for all subsequent experiments.

For the titration of SARS-CoV-2 after VBL exposure with and without antioxidants, the virus was titrated by performing fivefold serial dilutions of irradiated and control SNs in 96-well adhesion plates. The dilutions were used to infect pre-seeded VERO E6 cells at 70% confluence. After 72 h, cell viability was measured by the CellTiterGLO 2.0 assay (Promega, La Jolla, CA, USA). The viral titer was calculated using the improved Kärber method and expressed as TCID_50_/mL.

### Detection and quantification of SARS-CoV-2 RNA by qRT-PCR, Qubit assay, and NGS analysis

Viral RNA extraction was performed from the VBL-exposed and the CV SNs using a homemade guanidine thiocyanate-based (GuSCN) extraction method. Then, cDNA was generated using the LunaScript RT SuperMix Kit (New England Biolabs, MA, USA). qRT-PCR was performed using the Luna Universal Probe qPCR MasterMix (Euroclone, Pero, Italy) in a total volume of 20 µL containing 5 µL of cDNA, 10 µL of 2× Luna MasterMix, 10 pM each forward and reverse primer, 2.5 pM FAM-labeled probe and RNase-free sterile water to the final volume. The samples were then loaded on a Rotor-Gene Q instrument (Qiagen, Hilden, Germany) to detect the fluorescence intensity of the FAM-TaqMan probe in each sample, and results were analyzed using the Rotor-Gene Q 2.3.1 software. Each cDNA was quantified in duplicate, with primers and probes designed on the NC_045512.2 reference strain targeting the SARS-CoV-2 polybasic cleavage site in the spike region. To quantify viral RNA in culture SNs, a standard curve obtained by diluting a control plasmid that was previously quantified by ddPCR (65) was used in each qRT-PCR run to interpolate the cycle threshold (Ct) generated by SNs. Eventually, RNA concentration of the CV and of the VBL-treated virus was evaluated with fluorimetric Qubit assay (Qubit 4 Fluorometer) based on binding of RNA-selective fluorescent dyes, using the RNA Assay Kit (Life Technologies, Waltham, MA, USA).

The NGS analysis was carried out using the MiSeq system (Illumina, San Diego, CA, USA). The VBL-exposed and the CV SN-extracted RNAs were used to prepare the library for SARS-CoV-2 whole-genome sequencing by using the COVIDSeq Assay (Illumina, San Diego, CA, USA) and Artic V4.1 SARS-CoV-2 primers, following the manufacturer’s instructions. SARS-CoV-2 sequences were assembled with the DRAGEN COVID lineage software (version 4.0.6).

### Protein carbonyls labeling with DNPH

Purified SARS-CoV-2 (2019-nCoV) Nucleocapsid Protein (His tag), SARS-CoV-2 (2019-nCoV) Spike Protein (RBD, His Tag), SARS-CoV-2 (2019-nCoV) envelope (CoV-E) protein, and SARS-CoV-2 (2019-nCoV) Helicase-His Recombinant Protein were purchased (Sino Biological, Eschborn, Germany), to facilitate their individual characterization. Specifically, we aimed to determine whether exposure to light could induce oxidative damage using the DNPH assay.

The 2,4-dinitro-phenylhydrazin (DNPH) is a specific probe that reacts with protein carbonyl groups, forming protein-conjugated dinitrophenylhydrazones (DNP). These protein-DNP adducts are recognized by specific primary antibodies, allowing DNPH to be used for the derivatization of protein carbonyls and subsequent analysis by the Western Blot method. For each protein, 3 µg per condition were exposed to VBL in 1× PBS, 1:3 DMEM in PBS, and 1:20 DMEM in PBS at room temperature for 90 min. Untreated samples were kept under the same conditions, away from VBL, in Eppendorf tubes. The samples were derivatized by adding an equal volume of derivatization solution (DNPH 5 mM, TFA 5% vol/vol, and SDS 6% wt/vol) and incubated for 30 min at room temperature in the dark, vortexing every 10 min. An equal volume of neutralization solution (Tris 1 M, 30% glycerol, 2% beta-mercaptoethanol, and bromophenol blue traces) was added to stop the derivatization reaction. Finally, the samples were boiled for 5 min at 95°C and resolved by SDS-PAGE.

### SDS-PAGE and western blot

Immunoblot was performed according to a previous study (66). The samples were resolved by SDS-PAGE using 4–12% Bis-Tris gels (Invitrogen), transferred to PVDF membrane, and incubated overnight at 4°C with a primary anti-dinitrophenyl antibody (Merck KGaA, Darmstadt, Germany) diluted 1:10,000 in 3% milk-0.1% Triton X-100 in 1× PBS. The following day, membranes were washed three times in 3% milk-0.1% Triton X-100 in 1× PBS for 10 min each, then incubated for 2 h at room temperature with a secondary anti-rabbit-HRP conjugated antibody (Merck KGaA, Darmstadt, Germany) diluted 1:5,000 in 3% milk-0.1% Triton X-100 in 1× PBS. Membranes were washed three times in the same solution. In the end, membranes were washed for 15 min with Triton X-100 in 1× PBS and two times with Tris-HCl 0.05 M pH 6.8. The chemiluminescent signal was detected with Lite Up (Euroclone S.p.A., Milan, Italy) and captured with Imagine Quant Las 4000 (GE Healthcare, Milan, Italy). Ponceau red staining (SERVA Electrophoresis GmbH Heidelberg, Germany) was performed as a control (Fig. S2).

### Statistical analysis

All viral viability data collected during the study were entered into a database, including the following variables: test date, test identifier, TCID_50_/mL, virus species, and test inoculum concentrations. Descriptive statistics of the empirical data were calculated as the mean of the logarithmic reduction in base 10 (log_10_) of the TCID_50_/mL together with 95% confidence intervals to assess the reliability of the measurements. Microsoft Excel software (ver. 2021) was used to organize the database. Statistical analysis was performed with Stata software (ver. 17). For the immunofluorescence assay, statistical analyses were performed using Prism 9 software (GraphPad Software, La Jolla, CA, USA). Differences between VBL exposure and virus control were estimated using a two-way ANOVA. Values were expressed as the mean ± SD and *P* < 0.05 was considered statistically significant.

## Data Availability

The data that support the findings of this study are available from the corresponding authors upon request. Source data are provided with this paper and its supplemental material.

## SUPPLEMENTAL MATERIAL

Supplemental material aem.00403-25-s0001.docx Figures S1 and S2; Tables S1 to S3.

ASM does not own the copyrights to Supplemental Material that may be linked to, or accessed through, an article. The authors have granted ASM a non-exclusive, world-wide license to publish the Supplemental Material files. Please contact the corresponding author directly for reuse.
