# Supplementary material for: Analysis of the SARS-CoV-2 inactivation mechanism using violet-blue light (405 nm)
Source: Appl Environ Microbiol. 2025 May 14;91(6):e00403-25. doi: 10.1128/aem.00403-25 (PMC12175510; doi:10.1128/aem.00403-25)
Supplement: Supplemental material — Figures S1 and S2; Tables S1 to S3. [file aem.00403-25-s0001.docx]

**Analysis of the SARS-CoV-2 inactivation mechanism using violet-blue light (405 nm)**

by **Davide Amodeo**^1, *^, **Serena Marchi**^2^, **Lia Fiaschi**^1^, **Luisa Raucci**^3^, **Camilla Biba**^1^, **Valentina Salvestroni**^2^, **Claudia Maria Trombetta**^2^, **Ilaria Manini**^2^, **Maurizio Zazzi**^1^, **Emanuele Montomoli**^2,4^, **Ilaria Vicenti**^1^, **Gabriele Cevenini**^1^ and **Gabriele Messina**^2^

^1^Department of Medical Biotechnologies, University of Siena, Siena, Italy

^2^Department of Molecular and Developmental Medicine, University of Siena, Siena, Italy

^3^Department of Biotechnology, chemistry and pharmacy, University of Siena, Siena, Italy

^4^VisMederi Srl, Siena, Italy

*Corresponding author: davide.amodeo@dbm.unisi.it

**Supplementary information**

**Supplementary figure**


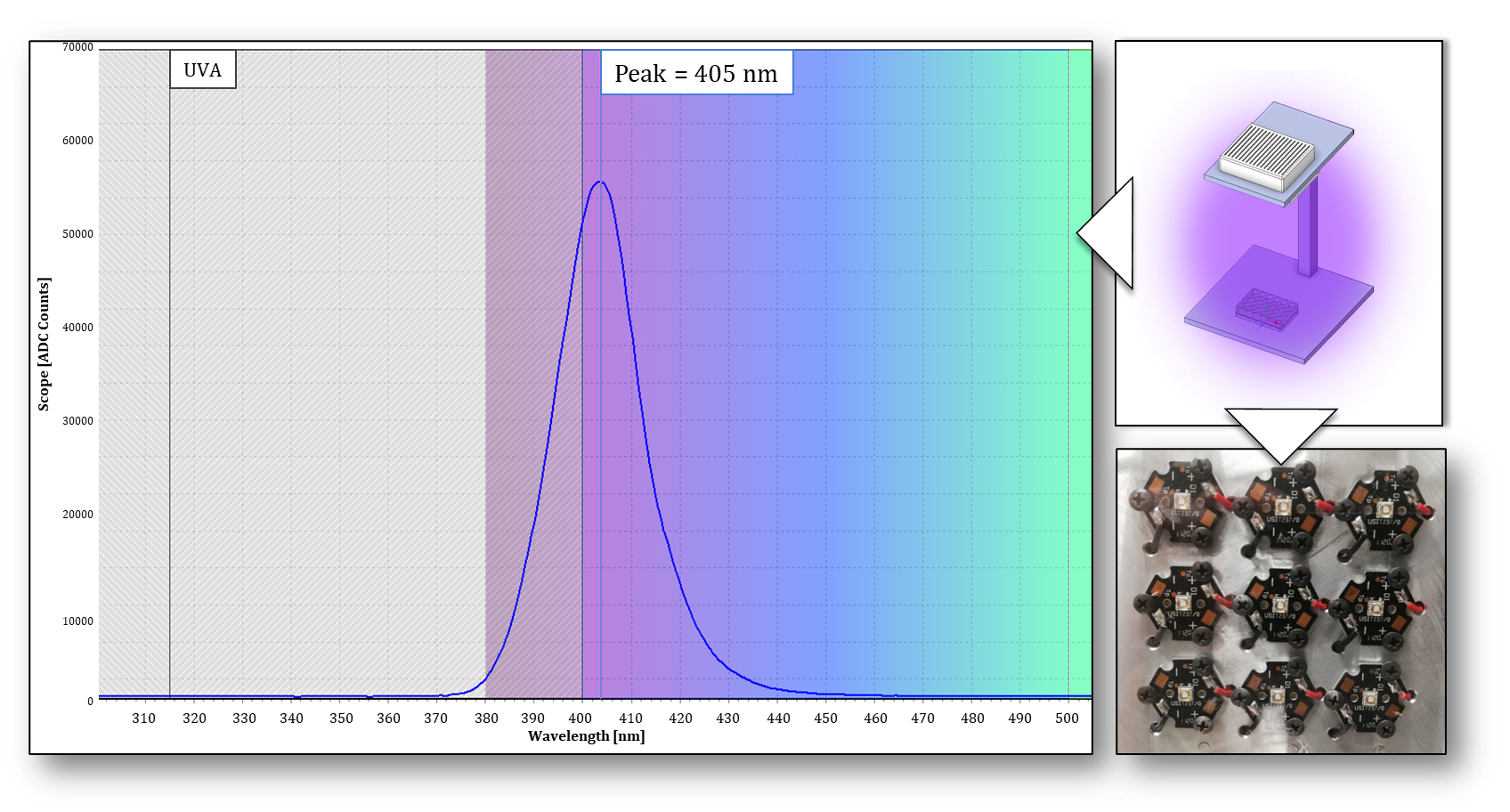


**Supplementary Fig. 1: VBL LED specifications and light spectrum characterisation.**

The picture shows the arrangement of the LED array (bottom right), model LITEON LTPL-C034UVG405-PA. The light spectrum emitted by the LEDs (left) was characterised using the spectrum of the Avantes ULS2048CL EVO photometer and processed using Avasoft analysis software. In the graph, Scope identifies the number of photons recorded during the measurement.


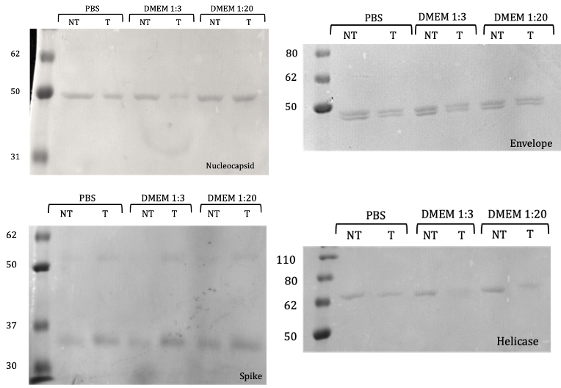


**Supplementary Fig. 2: SARS-CoV-2 proteins Ponceau red staining.**

The procedure was used to evaluate loaded proteins in presence (T) or absence (NT) of violet-blue light (405 nm) exposure. Before incubation with the blocking solution, PVDF membranes were stained with Ponceau Red to verify protein loading. The four proteins exhibit no signs of alteration, regardless of treatment.

**Supplementary table**

| **Supplementary Table 1: Infectious virus titre (TCID_50_/mL) of the VBL-treated and control virus at three dilutions in PBS.** The fold-change is expressed as the ratio of the control virus TCID_50_/mL to the VBL-treated virus TCID50/mL. The TCID50/mL % reduction refers to the VBL-treated virus compared to the control virus (CV). | | | | | | | | |
| --- | --- | --- | --- | --- | --- | --- | --- | --- |
| **SN dilution  factor** | **VBL-treated virus** | | | **Control virus** | | | **Fold-change** | **%  Reduction** |
|  | **TCID_50_/mL** | **C.I.   lower** | **C.I.   upper** | **TCID_50_/mL** | **C.I.   lower** | **C.I.  upper** |  |  |
| **1:3** | **2.08E+04** | 7.29E+03 | 5.91E+04 | **1.09E+09** | 4.13E+08 | 2.85E+09 | 52404 | 99.9981 |
| **1:20** | **2.78E+04** | 1.06E+04 | 7.29E+04 | **9.71E+08** | 2.47E+08 | 3.81E+09 | 34928 | 99.9971 |
| **1:1,000** | **2.08E+04** | 7.29E+03 | 5.91E+04 | **8.68E+06** | 2.49E+06 | 3.03E+07 | 417 | 99.7604 |

| **Supplementary Table 2: Infectious virus titer (log_10_ TCID_50_/mL) of all the titrated viruses at three dilution factors with and without the addition of NAC (0.5 and 0.05 mM), AsA (0.5 and 0.05 mM) and SOD (0.03 and 0.003 mM).** The Log_10_ reduction refers to the VBL-exposed virus plus antioxidants with respect to the control virus (CV), not exposed to the radiation but incubated for the same time (90 min) at the different antioxidant concentrations. | | | | | | |
| --- | --- | --- | --- | --- | --- | --- |
| **SN Dil. Factor** | **Antioxidant** | **CV** | **VBL-exposed virus** | | | **Log_10_  reduction** |
|  |  | Log_10_ TCID_50_/mL | Log_10_ TCID_50_/mL | lower C.I. | upper C.I. |  |
| **1:3** | 5 mM NAC | 9.833 | 5.890 | 5.297 | 6.484 | 3.943 |
|  | 0.5 mM NAC | 9.944 | 5.017 | 4.562 | 5.471 | 4.927 |
| **1:20** | 5 mM NAC | 9.083 | 5.493 | 4.898 | 6.086 | 3.590 |
|  | 0.5 mM NAC | 9.045 | 5.841 | 5.248 | 6.436 | 3.204 |
| **1:1,000** | 5 mM NAC | 6.393 | 4.841 | 4.248 | 5.436 | 1.551 |
|  | 0.5 mM NAC | 8.127 | 4.667 | 4.369 | 4.965 | 3.461 |
| **1:3** | 5 mM AsA | 9.771 | 6.415 | 5.628 | 7.201 | 3.356 |
|  | 0.5 mM AsA | 9.934 | 5.190 | 4.705 | 5.677 | 4.744 |
| **1:20** | 5 mM AsA | 8.996 | 6.540 | 5.735 | 7.346 | 2.455 |
|  | 0.5 mM AsA | 9.114 | 4.968 | 4.513 | 5.423 | 4.146 |
| **1:1,000** | 5 mM AsA | 6.403 | 4.667 | 4.152 | 5.182 | 1.737 |
|  | 0.5 mM AsA | 8.152 | 4.667 | 4.369 | 4.965 | 3.486 |
| **1:3** | 0.03 mM SOD | 9.732 | 3.270 | 2.971 | 3.567 | 6.463 |
|  | 0.003 mM SOD | 9.653 | 4.318 | 3.863 | 4.772 | 5.335 |
| **1:20** | 0.03 mM SOD | 8.987 | 4.093 | 3.675 | 4.513 | 4.893 |
|  | 0.003 mM SOD | 9.061 | 4.968 | 4.455 | 5.481 | 4.093 |
| **1:1,000** | 0.03 mM SOD | 6.706 | 5.190 | 4.705 | 5.677 | 1.516 |
|  | 0.003 mM SOD | 5.854 | 5.017 | 4.719 | 5.314 | 0.837 |

| **Supplementary Table 3: Set-up of assay to evaluate the effects of VBL exposure on viral replication.** VBL-treated and untreated virus was used to infect VERO E6 and the viral replication was determined at 24 and 48 hours post infection (P.I.) quantifying the nucleocapsid (N) protein by ELISA or at 72 hours P.I. quantifying the virus induced cytopathic effect (CPE) by ATP measurement. Results were expressed as the logarithm of TCID_50_/mL and relative Confidence Interval (C.I.) obtained using the improved Kärber method. | | | | | | | |
| --- | --- | --- | --- | --- | --- | --- | --- |
| **Readout Method** | **VBL-exposed virus** | | |  | **Control virus** | | |
|  | **(log_10_ TCID_50_/mL)** | **lower C.I.** | **upper C.I.** |  | **(log_10_ TCID_50_/mL)** | **lower C.I.** | **upper C.I.** |
| **ELISA 24h P.I.** | 3.10 | 2.76 | 3.44 |  | 5.55 | 5.13 | 5.97 |
| **ELISA 48h P.I.** | 3.45 | 3.45 | 3.45 |  | 6.78 | 6.16 | 7.40 |
| **CPE 72h P.I.** | 4.15 | 4.15 | 4.15 |  | 7.47 | 6.96 | 7.98 |
